# Genome-wide profiling and differential expression of microRNA in rat pluripotent stem cells

**DOI:** 10.1038/s41598-017-02632-0

**Published:** 2017-06-05

**Authors:** Vladimir V. Sherstyuk, Sergey P. Medvedev, Evgeniy A. Elisaphenko, Evgeniya A. Vaskova, Maxim T. Ri, Yuri V. Vyatkin, Olga V. Saik, Dmitry N. Shtokalo, Evgeniy A. Pokushalov, Suren M. Zakian

**Affiliations:** 1 Federal Research Center Institute of Cytology and Genetics, the Siberian Branch of the Russian Academy of Sciences, 10 Lavrentyeva Ave., Novosibirsk, 630090 Russia; 2 Siberian Federal Biomedical Research Center, Ministry of Healthcare of the Russian Federation, 15 Rechkunovskaya St., Novosibirsk, 630055 Russia; 3 Institute of Chemical Biology and Fundamental Medicine, the Siberian Branch of the Russian Academy of Sciences, 8 Lavrentyeva Ave., Novosibirsk, 630090 Russia; 4 Novosibirsk State University, 2 Pirogova St., Novosibirsk, 630090 Russia; 5 AcademGene LLC, 6 Lavrentyeva Ave., Novosibirsk, 630090 Russia; 6 St. Laurent Institute, 317 New Boston St., Woburn, MA 01801 USA; 7 A.P.Ershov Institute of Informatics Systems, 6 Lavrentyeva Ave., Novosibirsk, 630090 Russia

## Abstract

MicroRNAs (miRNAs) constitute a class of small noncoding RNAs that plays an important role in the post-transcriptional regulation of gene expression. Much evidence has demonstrated that miRNAs are involved in regulating the human and mouse pluripotency. Nevertheless, to our knowledge, miRNAs in the pluripotent stem cells of one of the most commonly used model organisms – the *Rattus norvegicus* have not been studied. In the present study, we performed deep sequencing of small RNA molecules in the embryonic fibroblasts, embryonic stem cells, and induced pluripotent stem cells of laboratory rats. Bioinformatics analysis revealed 674 known miRNAs and 394 novel miRNA candidates in all of the samples. Expression of known pluripotency-associated miRNAs, such as the miR-290–295 and miR-183-96-182 clusters as well as members of the miR-200 family, was detected in rat pluripotent stem cells. Analysis of the targets of differentially expressed known and novel miRNAs showed their involvement in the regulation of pluripotency and the reprogramming process in rats. Bioinformatics and systems biology approaches identified potential pathways that are regulated by these miRNAs. This study contributes to our understanding of miRNAs in the regulation of pluripotency and cell reprogramming in the laboratory rat.

## Introduction

Pluripotent stem cells (PSCs) are a unique model for studying early development, disease modelling, and use in regenerative medicine. PSCs are capable of self-renewing and differentiating into all cell types of the adult organism. Two types of pluripotent cells exist – embryonic stem cells (ESCs) and induced pluripotent stem cells (iPSCs). ESCs are derived from the inner cell mass of blastocysts, and iPSCs are generated from somatic cells by the overexpression of Yamanaka factors (Oct4, Sox2, Klf4 and c-Myc)^1–5^. PSC fate is regulated by a complex network including transcription factors, signalling pathways, epigenetic modifications, and non-coding RNAs^6–8^. The regulatory pathways of self-renewal and pluripotency are not completely understood, especially in rats. The laboratory rat is widely used in various biomedical research fields such as disease modelling, pharmacology and toxicology^9^. However, the absence of rat PSCs has, for a long time, limited its further application in biomedicine. Rat pluripotent cells were first derived in 2008 using special serum-free conditions, leukaemia inhibitory factor, and inhibitors of the MEK1/2 and GSK3 kinases^10, 11^. It is known that PSCs have species-specific properties and that microRNAs (miRNAs) play a substantial role in the regulation of pluripotency^12–15^.

miRNAs are small 18–23-nt non-coding RNAs that regulate gene expression by mRNA degradation or translation inhibition^16, 17^. miRNAs are critical for the regulation of self-renewal and pluripotency, as was shown by knockout studies of Dicer and DGCR8 – enzymes that are involved in miRNAs processing^18–21^. Some miRNAs, such as miR-291, miR-294 and miR-295, can enhance reprogramming that is induced by OSK factors^22^. Moreover, overexpression of a miR-302-367 cluster was sufficient to reprogramme human and mouse fibroblasts without overexpressing the Yamanaka factors^23^.

Previous genome-wide sequencing studies of rat miRNAs have been conducted using adult tissues. Rat miRNAs were sequenced and analysed from several neural compartments and cell lines, such as thyroid FRTL-5 cells and their differentiated derivatives^24^ as well as six different tissues, including spleen, liver, brain, testis, heart, kidneys^25^. Recently, miRNA analysis was performed on the 21 and 23 organs of male and female Sprague Dawley rats, respectively^26^. However, miRNA expression in rat PSCs has not been studied yet. We recently derived rat ESCs and iPSCs and characterized their transcriptional profile^27^. All of our derived ESC and iPSC lines were AP-positive, expressed key pluripotency markers, and were able to differentiate into derivatives of all three germ layers *in vitro* (embryoid bodies) and *in vivo* (teratoma test). Female iPSC lines are characterized by two active X-chromosomes. The rat ESCs and iPSCs were also able to contribute to the tissue development of chimeric organisms in the xenogenic rat-mouse model. In this study, we performed next generation sequencing of small RNA libraries from rat ESCs, iPSCs and embryonic fibroblasts (EFs). We analysed miRNA expression in these cell types and identified novel rat miRNAs that were expressed in PSCs and EFs. Using the KEGG signalling pathways and Gene Ontology (GO) enrichment analyses of known and novel miRNA targets, we revealed the involvement of miRNAs in the regulation of pluripotency and cell reprogramming. These findings provide new insights into pluripotency network regulation in rats.

## Materials and Methods

### Cell cultures

Rat EFs, ESCs and iPSCs were previously obtained and characterized in our lab^27^. EFs were isolated from E12 rat embryos (WAG and Brattleboro strains). ESCs were derived from the inner cell mass of E4.5 blastocysts as described previously^10, 11^. iPSCs were produced from rat EFs by the overexpression of *Oct4*, *Sox2*, *Klf4* and *Myc* using a lentiviral doxycycline-inducible system^28^. Cells were cultivated as described^27^. EFs were maintained in Dulbecco’s modified Eagle’s medium (DMEM) mixed with Ham’s F12 medium (1:1), containing 10% foetal bovine serum, GlutaMAX^TM^, 5,000 U/mL penicillin/streptomycin, nonessential amino acids, sodium pyruvate, and 0.1 mM 2-mercaptoethanol. ESCs and iPSCs were cultivated in N2B27 medium containing GlutaMAX^TM^, 5,000 U/mL penicillin/streptomycin, 0.1 mM 2-mercaptoethanol, 1,000 U/mL mouse LIF (StemRD), 1 µM PD0325901 (StemRD), and 3 µM CHIR99021 (StemRD). ESC and iPSC differentiation was performed for 24 days on tissue culture surfaces coated with gelatine according to a previously published protocol^27^. Cells were disaggregated with collagenase IV or TrypLE and were seeded on a gelatine-treated surface at a density of 10^4^ cells/cm^2^ in medium containing a 1:1 mixture of N2B27 (without LIF, PD0325901 or CHIR99021) and DMEM/F12 media supplemented with 10% foetal bovine serum, nonessential amino acids, GlutaMAX, 10 ng/mL basic fibroblast growth factor, and 10 mM Y27632 (R&D).

### Small RNA extraction, sequencing and qRT-PCR analysis

Cells were resuspended in RNAlater and used for small RNA extraction with the mirVana™ miRNA Isolation Kit (Ambion, #AM1560). RNA quality was analysed using an Agilent Bioanalyzer. A 50-ng quantity of small RNA was used to prepare a library, which was then sequenced on the Illumina GAIIX. Raw data were deposited into the SRA database with the accession numbers SRX395433-395442.

Small RNA for cDNA synthesis were extracted from cells and tissues with the mirVana™ miRNA Isolation Kit. cDNA was synthesized by the NCode™ VILO™ miRNA cDNA synthesis Kit (Invitrogen) from 1 µg of total RNA. Real-time PCR was performed using the HS-qPCR SYBR Blue mix (Biolabmix) with a custom miRNA-specific primer and a universal primer from Invitrogen on the LightCycler480 System (Roche). Reaction conditions were 95 °C for 5 min followed by 40 cycles of 95 °C for 15 secs and 60 °C for 30 sec. Reaction specificity was verified by melting curve analysis. Reactions were performed in triplicate. Sequences of the miRNA-specific primers are provided in Supplementary Table S1. Data were normalized to *Rnu1a* expression.

### Mapping and filtration of reads

Raw reads of 11 samples were processed with the FASTX Toolkit v0.0.14 in two steps. First, the data were trimmed with the fastq_quality_trimmer tool, leaving only reads with a tail quality of at least 30. Then, an adapter sequence was clipped with the fastx_clipper program, leaving only 15-nt reads or longer.

Filtered reads were mapped to the *R*. *norvegicus* reference genome (version rn5) from the UCSC Genome Browser^29–32^ combined with the ribosomal RNA reference for *R*. *norvegicus* from the Silva database^33^. The BWA v0.7.10-r806 software was utilized for gapped/ungapped alignment of single-ended reads. If a read could be mapped to several places in the reference, only one random place was chosen. The portion of reads that mapped solely to chr1-20,X varied from 94.2% to 97.4% in different samples.

To assess the quality of reads, a set of 486 genomic intervals of known premature miRNAs for *R*. *norvegicus* was downloaded from miRBase v21 (ftp://mirbase.org/pub/mirbase/CURRENT/genomes/rno.gff3)^34^. The absolute majority (99%) of reads that mapped to the genomic intervals of pre-miRNA had a length of 18–27 nt. To keep the focus of this study on miRNA, we filtered out reads that were shorter than 18 and longer than 27 nt because they likely represented short RNA species, which are different from the miRNA class. The rest of the reads were further filtered from the ones mapped onto parts of the genome that is annotated with rmsk repeats (http://hgdownload.soe.ucsc.edu/goldenPath/rn5/database/rmsk.txt.gz)^29^ of class “RNA” (including rRNA, scRNA, snRNA, tRNA, srpRNA, RNA, and MARNA). This filtration did not influence any reads that were mapped to known miRNAs. A final set of mapped and filtered reads was called *informative reads*. A summary is presented in Table 1.

**Table 1:** Amount of *informative reads* and known miRNA reads in all samples.

Cell type	Sample	*informative reads*	known miRNA reads	Portion of known miRNA reads
iPSC	SU3.1.1	2652510	945963	35,66%
iPSC	SU3.1.2	430889	154478	35,85%
iPSC	SU3.2	413721	185124	44,75%
iPSC	SU3.3	209961	97608	46,49%
iPSC	NF13	253724	132544	52,24%
iPSC	NF21	272430	135334	49,68%
iPSC	QV8	604002	268954	44,53%
ESC	RES27	788002	388023	49,24%
ESC	dB50	184668	56953	30,84%
EF	RNFM1	1482905	1212691	81,78%
EF	RNFF1	1568042	1170241	74,63%

### Novel miRNA discovery with the custom peaks2mirna method

Wig files were created from the genome maps of the *informative reads* for two separate groups of samples: PSCs and EFs. The wig files contained RPM (reads per million) expression values for each position on each chromosome. RPM is calculated as the number of reads overlapping a position in the genome divided by the total number of *informative reads* in a group of samples. Then, wig files were inspected for genome intervals with specific double-peak signals, as explained in Supplementary Figure S1, with a custom Perl script using the following parameters: (i) peak height of 0.9 RPM or higher; (ii) peak length of 18–50 nt; (iii) distance between peaks of 8–30 nt; and (iv) no peaks within 50 nt vicinity. These parameters were selected to maximize the number of detectable, expressed, and known rat miRBase pre-miRNAs (245 of 403, or 60.7%). Then, the following additional filters were applied to the 718 newly identified potential pre-miRNAs to retain only the high-quality candidates: (1) no overlap with the 486 known rat miRBase miRNAs, (2) no overlap with the RNA repeats from the UCSC rn5 rmsk.txt (http://hgdownload.soe.ucsc.edu/goldenPath/rn5/database/rmsk.txt.gz)^29^, and (3) RNAFold program^35^ predicted a hairpin structure. As a result, 73 potential pre-miRNAs remained. Following further analyses of sequence homology, some of them were found to belong to the snoRNA class and had to be analysed separately.

### Novel miRNA discovery with the miRanalyzer tool

The miRanalyzer program^36^ was applied to each sample and entire dataset of 11 samples. The *R*. *norvegicus* genome (assembly version rn4) was used. The default parameters were used except for the “Fast detection of novel microRNAs”, which was unmarked. For each sample and separately for entire dataset of 11 samples, potential miRNAs that were named “perfect Dicer” were selected from the predictions. Then, only “perfect Dicers” that did not intersect with genome elements (empty “genome elements” column) were selected. Potential miRNA coordinates in rn4 were converted to rn5 using the UCSC LiftOver tool and then intersected with refSeq genes (“refGene.txt” downloaded from UCSC on 14.04.15). Only the entries that did not overlap the refSeq genes in rn4 and rn5 were kept. If a potential miRNA was found in at least two samples, it was labelled as a “cluster” miRNA. The longest or the most strongly expressed isoform was selected as a representative of the cluster. Potential novel miRNAs that were found by miRanalyzer and were predicted^35^ to have a hairpin structure were filtered against known rat miRNAs (ftp://mirbase.org/pub/mirbase/CURRENT/genomes/rno.gff3)^34^ and RNA repeats (http://hgdownload.soe.ucsc.edu/goldenPath/rn5/database/rmsk.txt.gz)^29^. As a result, 13 potential pre-miRNAs remained.

### Novel miRNA discovery with the miRDeep2 tool

MiRDeep2 v2.0.0.7^37^ was launched on the collapsed genome (rn5) maps of *informative reads*. A training set of known mature miRNAs included *R*. *norvegicus* (ftp://mirbase.org/pub/mirbase/CURRENT/genomes/rno.gff3)^34^ and *Mus musculus* (ftp://mirbase.org/pub/mirbase/CURRENT/genomes/mmu.gff3)^34^ miRNA sequences acquired from miRBase v21. Additionally, a set of miRNA hairpin sequences was taken from miRBase v21 (ftp://mirbase.org/pub/mirbase/CURRENT/hairpin.fa.gz)^34^. Potential novel miRNAs found by mirDeep2 were filtered against known rat miRNAs (ftp://mirbase.org/pub/mirbase/CURRENT/genomes/rno.gff3)^34^ and overlapping RNA repeats (http://hgdownload.soe.ucsc.edu/goldenPath/rn5/database/rmsk.txt.gz)^29^, which resulted in a list of 315 potential pre-miRNAs.

### Search for homologs of novel miRNAs

We took advantage of the UCSC genome browser tracks of rat and other species’ genes, including miRNA, that were mapped to the rn6 genome. To find homologs of novel miRNAs, we intersected their genomic intervals with exons and introns of the ensGene track (last updated 14 Sept, 2015) and the xenoRefGene track (last updated June 19, 2016) (http://hgdownload.soe.ucsc.edu/goldenPath/rn6/database/)^29^, with consideration of the DNA strand.

### Calculation of digital gene expression (DGE) of known and novel miRNA

Coordinates in the rn5 genome of 832 mature miRNAs were downloaded from miRBase v21 (ftp://mirbase.org/pub/mirbase/CURRENT/genomes/rno.gff3)^34^. The number of *All* mapped reads overlapping each miRNA genome interval, with consideration of DNA strand, was calculated in each sample. If a read overlapped the border of an interval, it was counted as 1. The sum of overlapping reads served as a raw digital expression level for each miRNA. Alternatively, raw expression was calculated for the *informative reads* (a subset of *All* reads that were 18–27 nt in length without RNA-type repeats). The raw expression of known miRNAs calculated by an alternative method was only 1% less than the expression that was calculated based on *All* mapped reads. To be less stringent, we took the expression level that was based on *All* reads for downstream analyses. In order to account for the different initial sequencing depths of samples, the RPM normalization procedure was applied to the raw expression values:

1 $${{\bf{x}}}_{{\bf{i}}}^{{\bf{j}}}={{\bf{X}}}_{{\bf{i}}}^{{\bf{j}}}\ast {\bf{1}}{{\bf{0}}}^{{\bf{6}}}/{\bf{Informative}}\_{\bf{read}}{{\bf{s}}}_{{\bf{i}}},$$

where i = 1, …, 11 samples; j = 1, …, 832 miRNAs; ${{\rm{X}}}_{{\rm{i}}}^{{\rm{j}}}$ – raw expression; ${{\rm{x}}}_{{\rm{i}}}^{{\rm{j}}}$ – RPM expression; and Informative_reads_i_ – number of *informative reads* in sample i (Supplementary Table S2). The expression values of novel miRNAs, based on their genome coordinates, were calculated in a similar way as in the known miRNAs with three exceptions (Supplementary Tables S3 and S4). First, primary sequences of novel pre-miRNAs were mapped to the rn6 genome with the blastn program^38^. If genome intervals of the blastn hits of two different pre-miRNAs overlap, their expression values were summed together. It was done to account for the random locations in the genome for multimapped reads. Second, novel pre-miRNA intervals longer than 50 nt were split into two equal subintervals to mimic possible mature miRNAs in the 5′ and 3′ shoulders. Third, alternative DGE levels were computed using just *informative reads* to calculate the ${{\rm{X}}}_{{\rm{i}}}^{{\rm{j}}}$ in formula (1). Both types of DGE based on *All* and *informative reads* were used to further classify novel miRNAs to Tier 1 and Tier 2. Tier 1 contained the novel miRNA species that were most likely to be real.

### Classification of novel miRNAs into tiers

By default, all potential pre-miRNAs were located in Tier 1. For each item, the following quality scores were calculated in order to sort them into two different tiers. First, the ratio of the number of *informative reads* to the number of *All* mapped reads was used to assess the portion of 18–27 nt reads. If this ratio was less than 0.5, the novel RNA molecule was moved to Tier 2. If the ratio was between 0.5 and 0.85 and the difference between the counts of *All* and *informative reads* was higher than 3, the pre-miRNA was also moved to Tier 2. The second classification parameter was calculated for novel pre-miRNA candidates in which the genomic interval was 50 nt or longer. Such intervals were divided into left and right parts of equal length. The parameter was used to assess the level of expression of the stem loop. It was calculated as the ratio m/(l + r), where l and r are the number of reads overlapping the left and right parts of the interval, respectively (they share reads overlapping the internal border), and m is the number of reads overlapping the border between the left and right parts. If the ratio was higher than 0.05, the potential pre-miRNA was moved to Tier 2.

### Principal component analysis (PCA) of miRNA expression levels

RPM expression data of 832 known miRNAs, excluding 158 zero-expression entries, were used. Additionally, 304 novel miRNAs with unique sequences and not homologous to known miRNAs were used independently. Using the R statistical computing program (http://www.R-project.org/)^39^, data were centred around the mean expression values, scaled to unit-variance and then processed using PCA^40^.

### Differential expression analysis of miRNAs

ESC and iPSC groups were combined together (PSCs) and compared with EFs. P-values were calculated with the edgeR software^41^ for each of the 674 expressed rat miRNAs from miRBase, assuming an overdispersed Poisson model of raw (not RPM normalized) expression counts. Parameters of overdispersion were fitted by the software, taking into account the common dispersion, trended dispersion and tagwise dispersion of the miRNA expression values. A set of 227 miRNAs satisfied a p-value cut-off of 10^−4^, which corresponded to the 3.0 × 10^−4^ false discovery rate (Benjamini-Hochberg). Alternatively, ANOVA was also performed using the ‘aov’ function in R after replacing zero values with uniform random values from interval (0 to 0.1) and log2 transformation (Supplementary Table S2). However, p-values based on just two samples in one of the groups (EFs) might have little credit. To get over this problem, differentially expressed miRNAs were required to satisfy very strong fold-change conditions without considering the p-value. The ratio between the group averages was required to be at least 3. Additionally, miRNAs upregulated in the PSC group must have a minimum RPM expression in PSC samples that is 2 times (or 10 times in a parallel analysis) higher than its maximum level in the EFs samples. We called this the *extremum fold change* method. In the same way, miRNAs were defined as upregulated in EFs if their minimum RPM in EFs was 2 times (or 10 times) higher than its maximum level in PSCs, and additionally, they had to have at least three mapped reads in each of the EF samples. Only 219 miRNAs passed this procedure of selection, and they had an edgeR p-value of less than 0.01. Differentially expressed novel miRNAs were defined in the same way.

### Clustering according to miRNA expression levels

Expression values of zero were replaced with 0.25, which is below the minimum positive value. After that, log2 transformation was applied. The clustering of miRNAs and samples based on expression values was performed using the ‘pheatmap’ function in R using the default parameters.

### Search for target genes of differentially expressed miRBase miRNAs

Known rat, mouse and human miRNA-target interactions were downloaded from the MiRTarBase database^42^ (http://mirtarbase.mbc.nctu.edu.tw/php/download.php; Catalogued by Species: hsa_MTI.xlsx, mmu_MTI.xls, rno_MTI.xls; accessed on 26/07/2016). Lists of the targets of differentially expressed known miRNAs were obtained taking the miRNA names as the key. They were additionally filtered using gene expression data obtained from sequencing experiments that were performed on the same biological samples^27^. Upregulated targets were kept if the corresponding miRNA was downregulated, and downregulated targets were kept if the corresponding miRNA was upregulated (Supplementary Table S5).

### Search for the target genes of novel miRNAs

Mature sequences of novel miRNAs were analysed for targets in the 3′ UTRs of rat refSeq transcripts using the TargetSpy v1.1^43^, miRanda v3.3a^44^ and TargetScan v7.0^45^ tools. To reduce the false positive rate, only targets that were predicted by all three programs were accepted (Supplementary Table S6).

### KEGG and GO enrichment analysis

Lists of genes found to be targeted by miRNAs were subjected to KEGG and GO enrichment analysis with the DAVID 6.8 Beta online tool^46^ using the default parameters. All rat genes were used as the background, and alternatively, only differentially expressed genes^27^ were used.

## Results

### Small RNA sequencing

**Figure 1:**
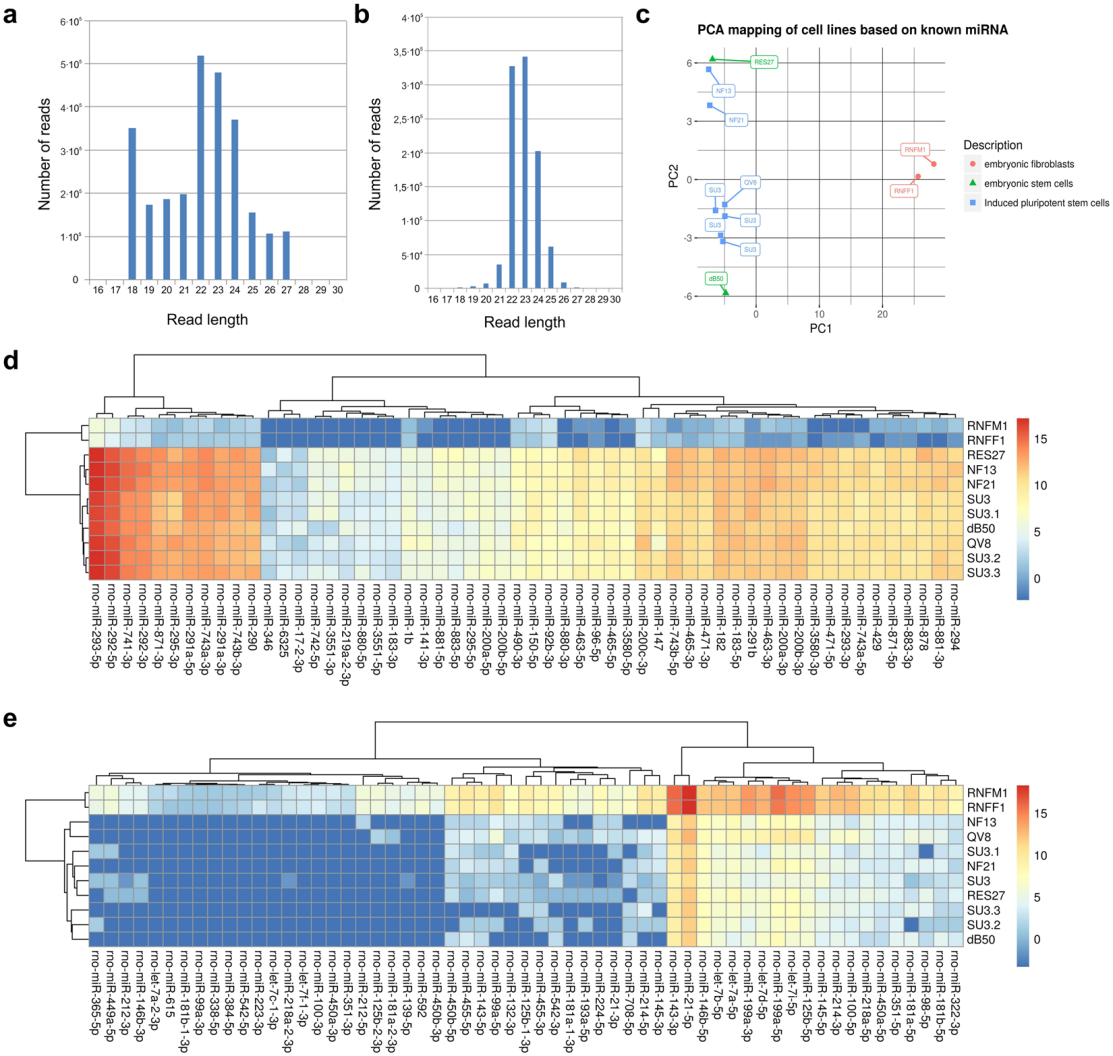
(**a**) The number of *informative reads* at different lengths for sample SU3. (**b**) The number of *informative reads* at different lengths for sample SU3 that were mapped to known rat miRNAs. (**c**) Principal component analysis of 11 samples based on RPM expression of 219 differentially expressed miRbase miRNAs. (**d** and **e**) Heatmaps of 56 upregulated miRNAs in PSCs (**d**) and 59 upregulated miRNAs in EFs (**e**). Known miRNAs with an *extremum fold change* more than 10, and the log2 transformation.

Small RNA libraries from two EF (RNFM1, RNFF1), two ESC (RES27, dB50), and seven iPSC samples (NF13, NF21, QV8, SU3, one technical, and two biological replicates) were generated and sequenced on the Illumina platform. Reads were filtered as described in the Materials and Methods section. A total of 32.9 million raw reads were generated by the sequencing of eleven libraries followed by trimming and adapter clipping. An absolute majority (31.9 million raw reads) mapped onto the reference genome, including 31.4 million reads that mapped on the chr1-20, X (*All* reads). After filtration against RNA-type repeats and after keeping only 18–27 nt sequences, a set of 8,860,854 *informative reads* was obtained. The exact amount of *informative reads* for each library is presented in Table 1. The length distribution of the *informative reads* was similar among different samples. Examples are given in Fig. 1a and b. Most of the *informative reads* were between 21–25 nt. To identify known miRNAs, coordinates of *All* reads that were mapped to the genome were compared with known miRNA coordinates from miRBase v21^34^. The portion of known miRNA reads (Table 1) varied from 30.84% (dB50) to 81.78% (RNFM1). In total, 674 known miRNAs were identified among all samples.

### Novel miRNA discovery

To discover novel miRNA species, the *informative reads* that were mapped to the rn5 genome were processed by three different methods: the custom peaks2mirna, miRanalyzer^36^ and miRDeep2^37^. Pre-miRNAs discovered by more than one method were collapsed by taking the maximum of their genomic intervals. Likely false positives, in which the primary sequence was repeated in the genome more than 15 times, were excluded. The number of repeats in the genome was calculated as the number of blastn^38^ hits, and the threshold was fitted to avoid false negatives. Finally, 388 potential novel pre-miRNAs including genomic duplicates were identified. This number included 39 pre-miRNAs that were homologous to rat or non-rat miRNAs and 40 molecules that were homologous to small nucleolar RNA (snoRNA). Homology analysis was done by overlapping genome intervals with the xenoRefGene and ensGene UCSC genome tracks^29^. This analysis allows nucleotide gaps, deletions and mismatches between homologs, including variance in miRNA seed sequence. The rest of the 309 candidates were split into Tier 1 and Tier 2 lists according to their quality scores as described in the Materials and Methods section and each had 248 and 61 items, respectively. Tier 1 and novel species that are homologous to known miRNAs are presented in Supplementary Table S3, which contains 394 mature miRNAs including genomic duplicates. Hereafter, this table will be referred to as Tier 1. Tier 2 and snoRNA homologs are presented in Supplementary Table S4, which includes 163 potential mature miRNAs including genomic duplicates. Hereafter, this table will be referred to as Tier 2. The two lists, in total, contain 557 potential novel miRNAs. Serial number in identificators of novel miRNA were assigned arbitrary and their order don’t imply to reflect importance or quality in both Supplementary Tables S3 and S4.

### Differential expression of miRNAs in rat pluripotent stem cells and embryonic fibroblasts

To reveal differentially expressed miRNAs, reads per million (RPM) gene expression was calculated for the 832 known and the 557 potential novel miRNAs using *All* mapped reads as described in the Materials and Methods section. Among the known miRNAs, 77 were found to be upregulated and 142 were found to be downregulated in PSCs compared to EFs, with at least a double *extremum fold change* (Supplementary Table S7). For novel Tier 1 miRNAs, the corresponding up- and downregulated genes were 10 and 13, respectively (Table 2). In Tier 2, there were 9 and 2, respectively (Supplementary Table S4). The criteria for miRNA up- or downregulation were described in the Materials and Methods section. Most of the miRNAs that were upregulated in PSCs have expression levels that are below the median in adult rat tissues, according to RT-PCR expression profiling with the TaqMan® Rodent miRNA card A v2.0 (GEO accession GSE66195, https://www.ncbi.nlm.nih.gov/geo/query/acc.cgi?acc=GSE66195). On the other hand, most of the downregulated miRNAs have high expression levels (above median) in somatic tissues in the same experiment (data not shown).

**Table 2:** Novel miRNA differentially expressed between PSCs and EFs.

Position (rn5)	Shoulder	**PSC** or EF	iPSC, RPM	ESC, RPM	EF, RPM	Homolog	miRdeep2	Custom	Pre-miRNA primary sequence
chr5:+:152026238–152026277	3p	EF > PSC	0	0	2.98		1	0	AAGGGGCAGGTTGCTGATACATTGTGACATAGACTTGGAAACAGTTGGTAATAGTGTACTGTGACCTGTCCTAGGGTGA
chr8:+:67350015–67350048	3p	EF > PSC	0	0	6.31		1	0	CTCAGACATGGTTCCTGTCCTCTTGGGTCTCAGTATTGATGTGGAGTACAGAGCACATGTTTGAGGGG
chr8:+:67395497–67395530	3p	EF > PSC	0	0	6.31		1	1	TCAGACATGGTTCCTGTCCTCTTGGGTCTCAGTATTGATGTGGAGTACAGAGCACATGTTTGAGGGG
chr5:+:83208017–83208048	5p	EF > PSC	0	0	3.65		1	1	TGGTCATGGTTCATGAACTGGAGCGGGTCCATCGTGTGTGTTCCAGTTCAACCTTGTCCCTGTT
chr3:−:124104501–124104531	5p	EF > PSC	0	0	3.26		1	0	CATGTTCCACTCACTCTCAGACGAAAACAGAGAGGCTCTGGGAGAGGTGTGGGACCAGTAC
chr10:−:32125896–32125927	5p	EF > PSC	0	0	2.64		1	0	TCTGTGAGTCTGAAGCCCACCTGGTTTACATATCAAGTTCCAAGTGCGCCAGACCCACATAGTG
chr6:−:137837930−137837960	5p	EF > PSC	2.34	0.63	15.73		1	0	TCTGCTCCTGCTCTTTCTGCTGCTGCTGTTCCTGTTGCCAGCGGGCGAGGAGGAGCGCAGCT
chr2:+:217974496–217974523	5p	PSC > EF	17.74	17.01	2.66		1	0	ATTGCCTGGTTTAGTCTCTGCTACTTGCAAGTACCAGGTCACTAAAACAGGCAGGG
chrX:−:151288045–151288072	3p	PSC > EF	11656.24	11023.61	3.90		1	0	TACTCAGATTAGTGTCACTCCAGGACATAAATATGTATGGCGCTCCTCTGAGTAGAA
chr14:+:26226537–26226565	5p	PSC > EF	61.89	72.76	0		1	1	TGCTTGCCTGGATTACAGTGAAGGTATTCATGTTTCACTGCACTCTAGACAGGCATTA
chrX:−:151288073–151288101	5p	PSC > EF	107.52	169.08	0		1	0	TACTCAGATTAGTGTCACTCCAGGACATAAATATGTATGGCGCTCCTCTGAGTAGAA
chr1:−:63523357–63523389	3p	PSC > EF	3395.86	3010.64	0.97	MIR372	1	1	CACTCAAATGTAGGAGCGCTCTTCTGATCTGGTTTAAGTGGAAAGTGTTGCATCGTTTGGGTGTCAT
chr1:−:63523390–63523423	5p	PSC > EF	243.23	199.24	0.33	MIR372	1	1	CACTCAAATGTAGGAGCGCTCTTCTGATCTGGTTTAAGTGGAAAGTGTTGCATCGTTTGGGTGTCAT
chr2:+:251220594–251220625	3p	PSC > EF	45.96	47.38	0	MIR302B	1	0	CCACTTTAACATGGGAATGCTTTCTGTCACATTGAAGAGTAAGTGCTGCCATGTTTTAGTAGA
chr2:+:251220844–251220873	3p	PSC > EF	37.22	30.67	0	MIR302	1	1	TAAACGTGGTTGTCCTTGCTTTGGAACGAAGAAAGTAAGTGCTTCCATGTTTGGGTGAT
chr1:+:148620388–148620416	5p	PSC > EF	95.17	32.11	0.97	MIR147B	1	0	GTGGAAACACTTCTGCACAAACTCGATTTTGATGCCAGTGTGCAGAAATGCTTCTGCT
chrX:−:140167614–140167645	5p	PSC > EF	18.99	31.98	0.67	MIR18B	0	1	TAAGGTGCATCTAGTGCTGTTAGTGAAGCAGCTTATAATCTACTGCCCTAAATGCCCCTTCTCG
chr7:+:70463555–70463594	5p	EF > PSC	2640.99	2751.3	9939.24	MIR26	0	1	GAGAGCCGGCTGTGGCTGGATTCAAGTAATCCAGGATAGGCTGTTTCCATCTGTGAGGCCTATTCTTGATTACTTGTTTCT
chr7:+:70463595–70463635	3p	EF > PSC	2609.2	2734.29	9854.73	MIR26	0	1	GAGAGCCGGCTGTGGCTGGATTCAAGTAATCCAGGATAGGCTGTTTCCATCTGTGAGGCCTATTCTTGATTACTTGTTTCT
chr3:+:16697079–16697110	5p	EF > PSC	8.53	7.95	2241.76	MIR199	0	1	CCCAGTGTTTAGACTACCTGTTCAGGACTCCCAAATTGTACAGTAGTCTGCACATTGGTTAGGCT
chr3:+:16697111–16697143	3p	EF > PSC	81.95	73.78	14456.4	MIR199	0	1	CCCAGTGTTTAGACTACCTGTTCAGGACTCCCAAATTGTACAGTAGTCTGCACATTGGTTAGGCT
chrX:+:105464335–105464360	5p	EF > PSC	0	0	3.3	Mir7093	1	0	TTTCCATCTGTCACCCTGCAGGTCTGCTGTAGCAGGTGGCACTGCTTGAAGCA
chr14:−:30139894–30139924	3p	EF > PSC	1.74	0	74.28	MIR365	0	1	GGGACTTTTGGGGGCAGATGTGTTTCCATTCCACTATCATAATGCCCCTAAAAATCCTTATTG

PCA was performed based on the RPM values of 219 differentially expressed known miRNAs. Figure 1c shows the PCA projection of eleven cell lines onto the first two principal components (PC). The first PC perfectly separated EF samples from the others. The second PC divided samples by gender into two groups: NF13, NF21 and RES27 were female and SU3, QV8 and dB50 were male. PC1 explained 81.1% while PC2 explained 6.6% of the total variance. PC1 also perfectly separated PSCs from EFs if considering all (not just differentially expressed) known or novel miRNAs (data not shown). We then performed sample clustering based on differentially expressed miRBase miRNAs (*extremum fold change* of at least 10) and on all expressed miRNAs. Figure 1d and e show heat maps of the clusters of miRNAs that are overexpressed in PSCs and EFs, respectively. There was no significant difference between the results of the hierarchical clustering of samples performed on all miRNAs and on only differentially expressed miRNAs (data not shown). Both methods, PCA and hierarchical clustering, revealed that there are principal differences in miRNA expression between EFs and PSCs but no major differences between ESCs and iPSCs.

A number of miRNAs that were found to be upregulated in rat PSCs, such as the miR-290-295 cluster and the miR-200 family miRNAs, are involved in pluripotency induction and maintenance in mice^21, 47^. Several miRNAs, such as members of the miR-183-96-182 cluster, miR-741, miR-881, miR-878, miR-743a, miR-743b, miR-465, miR-871, miR-880, are known to be expressed in mouse ESCs and were also expressed in rat PSCs; however, their function in pluripotency induction and regulation has not been directly shown^48–50^.

### GO and pathway analyses of targets of the differentially expressed miRNAs

#### Analysis of known miRNA targets

**Figure 2:**
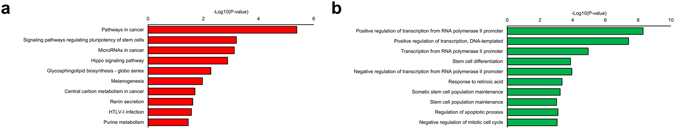
KEGG signalling pathway (**a**) and GO (**b**) enrichment analyses of the published targets of differentially expressed known miRNAs. The top 10 KEGG signalling pathways and 10 GO terms with the most significant p-value.

To reveal the function of differentially expressed miRNAs in rat PSCs and EFs, we performed an analysis of the published targets of these miRNAs in humans, mice, rats, and other species. We took known miRNAs with an *extremum fold change* of more than 10 between EFs and PSCs. Among them, 58 miRNAs were upregulated in the PSCs and 69 were upregulated in the EFs (Supplementary Table S7). Known miRNA targets were found using information from the MiRTarBase database^42^. Additionally, the targets were required to be downregulated for upregulated miRNAs and upregulated for downregulated miRNAs, which is consistent with the expected miRNA function. By this method, we found 388 published target genes for miRNAs that were upregulated in PSCs and 69 published targets for downregulated miRNAs (Supplementary Table S5). All of the up- and downregulated target genes were analysed with the DAVID^46^ web application tool to identify the associated KEGG pathways and GO terms. KEGG pathways enriched with target genes, using all rat genes as a background, are presented in Fig. 2a and in Supplementary Table S8. Most of the target genes were associated with cancer pathways, stem cell pluripotency regulatory pathways, and the Hippo signalling pathway. Of note is that the results persist if only differentially expressed genes were used as a background (data not shown). Among the enriched GO terms, stem cell differentiation, response to retinoic acid, somatic stem cell population maintenance, regulation of apoptotic process were present (Fig. 2b). The obtained results suggest that these differentially expressed miRNAs play a substantial role in the regulation of pluripotency and cell reprogramming in rats.

#### Analysis of novel miRNA putative targets

**Figure 3:**
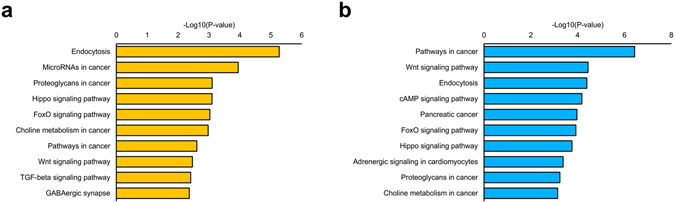
KEGG signalling pathway analysis of putative targets of (**a**) the top 10 highly expressed novel miRNAs in the PSC group and (**b**) the top 10 highly expressed novel miRNAs in the EF group.

Targets of 304 novel miRNAs with unique sequences that are not homologous to known miRNAs were predicted by TargetSpy v1.1^43^, miRanda v3.3a^44^, and TargetScan v7.0^45^ as described in the Materials and Methods section. In total 8,437 transcript targets were identified for novel miRNAs (Supplementary Table S6). GO enrichment and KEGG signalling pathway analyses were performed using the DAVID tool^46^ on the targets of the top 10 highly expressed novel miRNAs in the PSC group and the top 10 highly expressed novel miRNAs in the EF group, including all rat genes as a background. The results are presented in Fig. 3 and Supplementary Table S9. According to the GO annotations, the predicted target genes of novel miRNAs expressed in PSCs are related to processes of embryonic development, cell migration, signal transduction and gene expression regulation. According to KEGG annotation, putative targets are involved in endocytosis, pathways in cancer and the Hippo, FoxO, Wnt, and TGF-beta signalling pathways (Fig. 3a,b). The two latter networks are known to be involved in cell reprogramming and the regulation of pluripotency in rats^12, 47^.

### Validation of novel miRNAs by qRT-PCR

**Figure 4:**
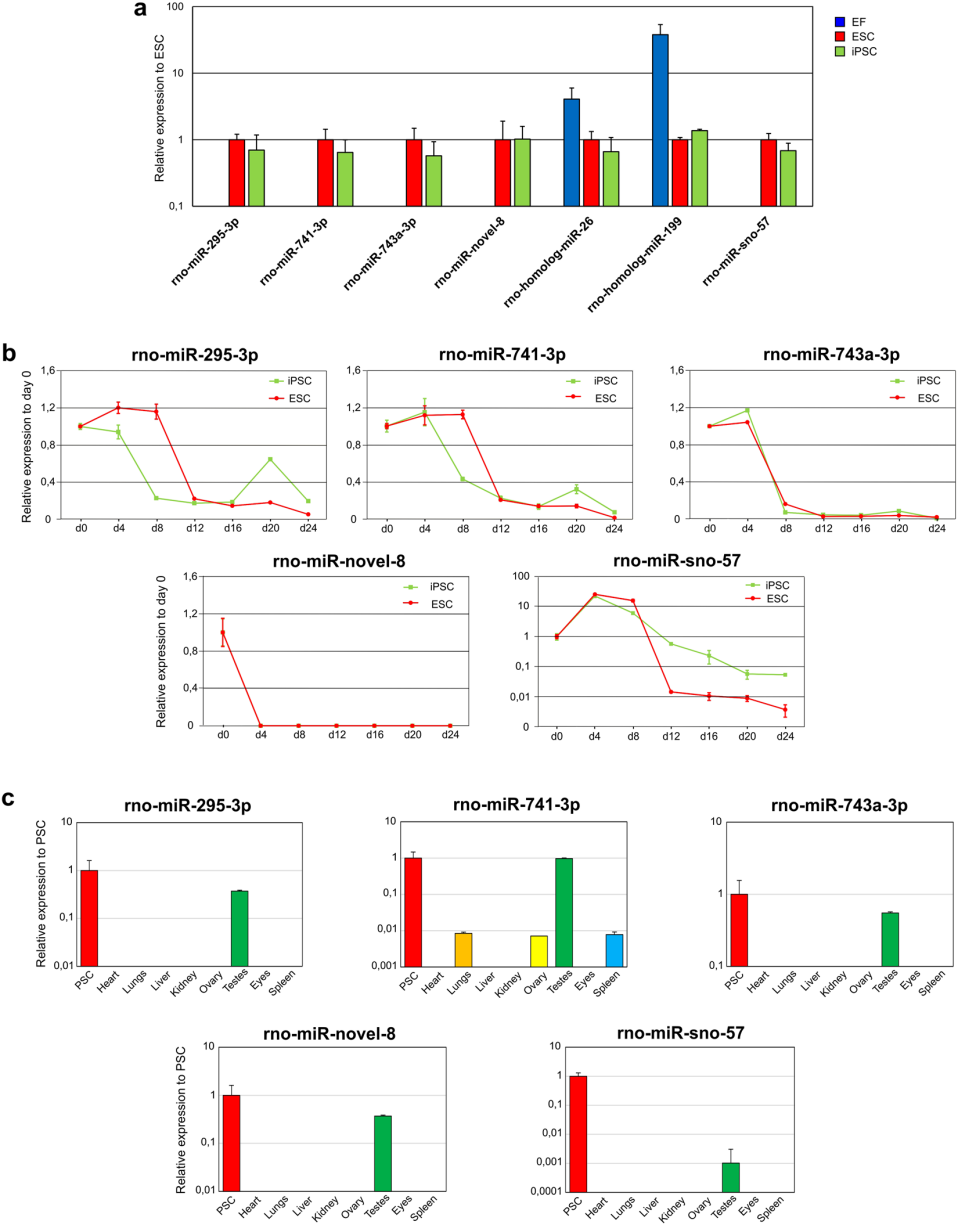
qRT-PCR analysis of known and novel miRNAs in (**a**) iPSCs, ESCs and EFs (**b**) during the differentiation of ESCs and iPSCs and (**c**) in rat adult organs.

To validate some of the novel miRNAs, we performed qRT-PCR analysis using the NCode™ system in the EF and PSC lines. Four novel miRNAs with the following coordinates: chrX:−:151288045–151288101 (rno-miR-novel-8); chr7:+:70463555–70463594 (rno-homolog-miR-26); chr3:+:16697111–16697143 (rno-homolog-miR-199); and chr18:−:69422790–69422857 (rno-miR-sno-57) were selected for the validation. rno-miR-novel-8, rno-homolog-miR-26, and rno-homolog-miR-199 miRNAs were selected from Tier 1, and rno-miR-sno-57 miRNA was selected from Tier 2. In addition, we analysed the expression of miR-741-3p and miR-743a-3p and found that, in accordance with sequencing data, they were highly expressed in rat PSCs. A pluripotency-associated miRNA, miR-295-3p, was used as a positive control. Expression of miR-295-3p was observed in rat PSCs and absent in rat EFs, which was consistent with its expression pattern in the mouse^51^ (Fig. 4a). qRT-PCR analysis demonstrated that the rno-miR-novel-8 and rno-miR-sno-57 miRNAs as well as the known miRNAs miR-741-3p and miR-743a-3p were expressed in rat PSCs and absent in rat EFs (Fig. 4a). rno-homolog-miR-26 and rno-homolog-miR-199 miRNAs were expressed in EFs, ESCs and iPSCs, which is consistent with the data obtained from sequencing. We also analysed the expression of the rno-miR-novel-8 miRNA, rno-miR-sno-57 miRNA, miR-741-3p, and miR-743a-3p, which are upregulated in PSCs during differentiation. We induced spontaneous differentiation of rat ESCs and iPSCs in a monolayer and analysed the expression of the miRNAs at various time points. We found that rat miR-295-3p, miR-741-3p and miR-743a-3p expression was significantly decreased during ESC and iPSC differentiation (Fig. 4b). For novel miRNAs, the expression of rno-miR-novel-8 miRNA was absent at the first time point (day 4) and subsequent time points of differentiation, whereas the expression of rno-miR-sno-57 miRNA increased at the first stages of differentiation and then decreased to levels lower than that in PSCs (Fig. 4b). We also assessed the expression of rno-miR-novel-8 miRNA, rno-miR-sno-57 miRNA, and the known miRNAs in different rat adult organs, including the heart, lungs, liver, kidneys, ovaries, testes, eyes and spleen. We found that miR-741-3p, miR-743a-3p, and rno-miR-novel-8 miRNA were expressed in the testis at levels comparable to that in PSCs. miR-741-3p was also expressed in the lungs, ovaries and spleen but at lower levels (Fig. 4c).

## Discussion

*R*. *norvegicus* is one of the most promising models for disease modelling and translational biomedicine. Pluripotent stem cells are currently widely used to generate various disease models^52^. An understanding of pluripotency and self-renewal regulation is required to improve the approaches to PSCs generation and cultivation, which will help realize the full potential of their application. Although much progress has been made in the understanding of the pluripotency regulatory network, it has not been fully investigated yet, especially in rats. miRNAs are known to play substantial roles in the regulation of pluripotency and self-renewal^14^. To study miRNA expression in rat PSCs and to find novel rat miRNAs, we performed genome-wide sequencing of small RNA libraries from rat ESCs, iPSCs, and EFs. Analysis of the sequencing data revealed the expression of 674 known miRNAs across all samples. Differential expression analysis of known miRNAs demonstrated that 77 miRNAs were upregulated and 142 miRNAs were downregulated in rat PSCs compared to EFs. Members of the miR-290-295 cluster (miR-290, miR-291a, miR-292, miR-291b, miR-293, miR-294 and miR-295-1) were the most abundant among known miRNAs that were upregulated in rat PSCs. It is known that mouse miR-290-295 and its human homologues in the miR-372 clusters are ESC specific and regulate cell cycle progression^20, 21^. Among the miRNAs upregulated in rat PSCs, we also identified miR-205 and members of the miR-200 family (miR-200a, miR-200b, miR-200c, miR-141 and miR-429), which promote mesenchymal to epithelial transition (MET) in mouse cells, a key step in fibroblast reprogramming^47^. Members of the miR-200 family are known to suppress the Zeb2 transcription factor, and its inhibition promotes MET^53^. Another miRNA cluster, miR-183-96-182, is known to be expressed in mouse PSCs^49^ and is also expressed in rat PSCs. The microRNA-183-96-182 cluster inhibits epithelial to mesenchymal transition (EMT) in cancer cells, and thus, the miRNA cluster may facilitate the reprogramming process by promoting MET^54–57^. Members of the miR-106-363 and miR-106b-25 clusters, which were shown to be upregulated during fibroblast reprogramming in mice^58^, were also upregulated in rat PSCs. Most of the miRNAs upregulated in rat PSCs are also expressed in mouse ESCs or iPSCs^48^. In addition, we found several rat miRNAs (including miR-3580, miR-3551, and miR-6325) whose expression had not been established in PSCs before.

A cluster of miRNAs, comprised of miR-463, miR-465, miR-471, miR-741, miR-743a, miR-743b, miR-871, miR-878, miR-880, miR-881, and miR-883, was located on the X chromosome and was expressed at a high level in rat PSCs according to the sequencing data. These miRNAs are known to be expressed in mouse ESCs and testes^48, 50, 59^. These miRNAs are testis specific in rats, according to the miRNA sequencing data of 21 male rat organs^26^. Using qPCR, we have shown that members of the miR-741-3p and miR-743a-3p clusters were expressed at high levels in rat ESCs and iPSCs and were downregulated during differentiation. These miRNAs are also expressed in some adult rat tissues, according to our data and RT-PCR expression profiling with the TaqMan® Rodent miRNA card A v2.0 (GEO accession GSE66195, https://www.ncbi.nlm.nih.gov/geo/query/acc.cgi?acc=GSE66195).

The number of known rat miRNAs is much lower compared to that in humans and mice. In this study, 394 novel mature miRNAs were identified using three methods – mirDeep2, miRanalyzer, and custom peaks2mirna. Most of the novel miRNAs were identified by the mirDeep2 method. In rat PSCs and EFs, 23 novel miRNAs were differentially expressed. Among them, 10 novel miRNAs were upregulated and 13 were downregulated in PSCs. This list includes homologues of human miR-302a and miR-302b. We found that these miRNAs were upregulated in rat PSCs. We also found a homologue of human miR-367. The human and mouse miR-302-367 cluster has been demonstrated to play an important role in the regulation of pluripotency. Moreover, overexpression of the miRNA cluster was sufficient to reprogramme fibroblasts to iPSCs^21, 23, 60^. However, expression levels of miR-302a and miR-302b were low in rat PSCs compared to the ESC specific miR-290-295 cluster. This is in agreement with the fact that high expression levels of miR-302 cluster are typical of mouse epiblast stem cells^48^. We also found a homologue of human mir-372 located inside the rat miR-290-295 cluster. miR-372 in humans is known to be connected to fibroblasts reprogramming, particularly by promoting MET^60^.

Four novel miRNAs were validated using qRT-PCR analysis. Three miRNAs were selected from Tier 1, and one was selected from Tier 2. Expression of the two validated novel miRNAs was observed in rat PSCs and was absent in EFs. Expression of rno-miR-novel-8 miRNA decreased during differentiation and was not observed in the analysed adult organs, except in the testes. The pattern is similar to ESC-specific miRNAs, such as members of the miR-290-295 cluster^51^. In addition, we found that the rno-miR-novel-8 miRNA was located less than 10 kb from miR-871, mir-3580 and miR-465, which were upregulated in rat PSCs. This may be a cluster of miRNAs that is involved in the regulation of pluripotency, and further investigations are required to shed light on its function.

miRNAs implement their functions through repressing target gene expression. In human and mouse PSCs, clusters of pluripotency-associated miRNAs control cell proliferation and prevent differentiation^58, 61^. To better understand the functions of known rat miRNAs that are differentially expressed between PSCs and EFs, we analysed their target genes that have been validated in rats, humans or mice. An additional criterion was that the change in the PSC and EF target gene expression levels had to be opposite of that for miRNAs. This criterion helped to improve the analysis of miRNA functions in the rat. In total, 388 downregulated targets were found for the upregulated miRNAs in PSCs, and 69 upregulated targets were observed for miRNAs that were downregulated in PSCs. Then, we performed KEGG and GO enrichment analyses of the target genes. The most common GO terms were related to transcriptional regulation, which indicated that a substantial part of the target genes functioned as transcriptional factors. Many GO terms, such as stem cell differentiation and response to retinoic acid, were related to cell differentiation, which was consistent with the function of miRNAs in suppressing PSCs differentiation^61, 62^. Self-renewal and differentiation processes are balanced in PSCs, and miRNAs are involved in the regulation of this process^58, 61^. We also found a stem cell population maintenance category that was relevant for targets of miRNAs that were differentially expressed in PSCs. Regulation of the apoptotic process was also an important category for pluripotent cells, because inhibition of apoptosis promotes pluripotency^63^ and was demonstrated for the ESC-specific miR-290-295 cluster^64^. We found enrichment of differentially expressed miRNAs and their targets in the KEGG signalling pathway, the stem cell pluripotency regulatory pathway, pathways in cancer, and the Hippo signalling pathway. Pathways in cancer are relevant for miRNAs that are upregulated in PSCs. It is known that miRNAs that are expressed in PSCs are frequently related to different types of cancer. The human miR-372-373 cluster promotes tumorigenesis, for example^65^. The Hippo signalling pathway represents a barrier for reprogramming, and its inhibition by LATS2 knockdown increases the efficiency of iPSC generation^66^. Analysis of the putative target genes of novel rat miRNAs has shown that the miRNAs may take part in the regulation of development and differentiation. In addition, KEGG signalling pathway analysis suggests the involvement of miRNAs in the regulation of the Wnt signalling pathway, which regulates rat PSC maintenance, and the TGF-beta pathway, which participates in the regulation of cell reprogramming^12, 47^. Based on the results, we suggest that the rat miRNAs identified in this study are involved in the regulation of pluripotency and reprogramming in rats.

In conclusion, we first compared the miRNA expression in rat embryonic stem cells and induced pluripotent stem cells with rat embryonic fibroblasts used for reprogramming. The miRNA expression signature in rat PSCs was similar to that in mouse PSCs, and most mouse pluripotency-associated miRNAs were also found in rats. A number of novel rat miRNAs were identified. Overall, our results shed light on the role of miRNAs in the pluripotency regulatory network of the laboratory rat.

## Electronic supplementary material

Supplementary Information

Supplementary Table S1

Supplementary Table S2

Supplementary Table S3

Supplementary Table S4

Supplementary Table S5

Supplementary Table S6

Supplementary Table S7

Supplementary Table S8

Supplementary Table S9
