# Supplementary material for: Genome-wide profiling and differential expression of microRNA in rat pluripotent stem cells
Source: Sci Rep. 2017 Jun 5;7:2787. doi: 10.1038/s41598-017-02632-0 (PMC5459850; doi:10.1038/s41598-017-02632-0)
Supplement: Supplementary file 1 — Supplementary Information [file 41598_2017_2632_MOESM1_ESM.pdf]

Genome-wide profiling and differential expression of microRNA in rat pluripotent stem cells

Vladimir V. Sherstyuk[1,2,3,4], Sergey P. Medvedev[1,2,3,4], Evgeniy A. Elisaphenko[1,2,3], Evgeniya A. Vaskova[1,2,3], Maxim T. Ri[5,6], Yuri V. Vyatkin[1,4,5,6], Olga V. Saik[1], Dmitry N. Shtokalo[1,5,6,7], Evgeniy A. Pokushalov[2], Suren M. Zakian[1,2,3,4,*]

[1]Federal Research Center Institute of Cytology and Genetics, the Siberian Branch of the Russian Academy of Sciences, 10 Lavrentyeva Ave., Novosibirsk, 630090, Russia;

[2]Siberian Federal Biomedical Research Center, Ministry of Healthcare of the Russian Federation, 15 Rechkunovskaya St., Novosibirsk, 630055, Russia;

[3]Institute of Chemical Biology and Fundamental Medicine, the Siberian Branch of the Russian Academy of Sciences, 8 Lavrentyeva Ave., Novosibirsk, 630090, Russia;

[4]Novosibirsk State University, 2 Pirogova St., Novosibirsk, 630090, Russia;

[5]AcademGene LLC, 6 Lavrentyeva Ave., Novosibirsk, 630090, Russia;

[6]St. Laurent Institute, 317 New Boston St., Woburn, MA 01801, USA;

[7]A.P.Ershov Institute of Informatics Systems, 6 Lavrentyeva Ave., Novosibirsk, 630090, Russia;

* Corresponding author, e-mail: zakian@bionet.nsc.ru

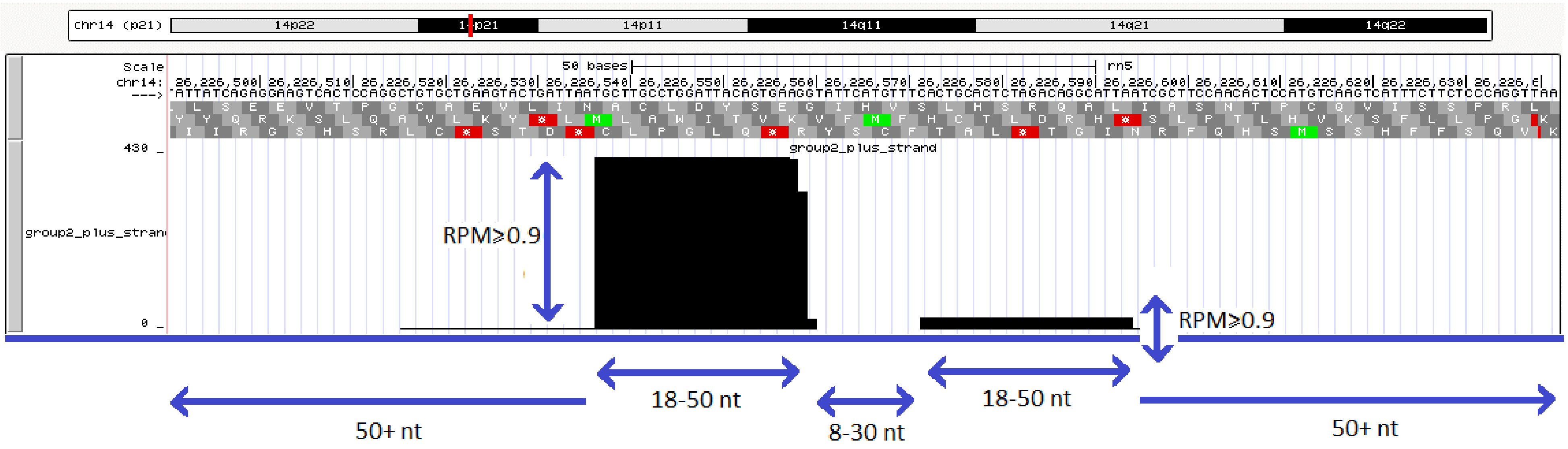

Figure S1 Double-peak method scheme for miRNA discovery

Following parameters were used to detect miRNAs: (i) – peaks height 0.9 RPM or higher, (ii) – peaks length 18-50 nt, (iii) – distance between peaks 8-30 nt, (iv) – distance to the nearest other peaks 50 nt or more.
